# Distributional patterns of item responses and total scores on the PHQ-9 in the general population: data from the National Health and Nutrition Examination Survey

**DOI:** 10.1186/s12888-018-1696-9

**Published:** 2018-04-23

**Authors:** Shinichiro Tomitaka, Yohei Kawasaki, Kazuki Ide, Maiko Akutagawa, Hiroshi Yamada, Yutaka Ono, Toshiaki A. Furukawa

**Affiliations:** 1 Department of Mental Health, Panasonic Health Center, Landic building 3F, Nishishinbashi 3-8-3, Minato-ku, Tokyo, 105-0003 Japan; 2 Department of Drug Evaluation and Informatics, School of Pharmaceutical Sciences, University of Shizuoka, 52-1 Yada, Suruga-ku, Shizuoka, 422-8526 Japan; 3 Department of Health Promotion and Human Behavior, Department of Clinical Epidemiology, Kyoto University Graduate School of Medicine/School of Public Health, Yoshida Konoe-cho, Sakyo-ku, Kyoto, 606-8501 Japan; 4 Clinical Research Center, Chiba University Hospital, 1-8-1, Cho-ku, Chiba-shi, 260-8677 Japan; 5 Department of Pharmacoepidemiology, Graduate School of Medicine and Public Health, Kyoto University, Yoshida Konoe-cho, Sakyo-ku, Kyoto, 606-8501 Japan; 6 Center for the Promotion of Interdisciplinary Education and Research, Kyoto University, Yoshida-honmachi, Sakyo-ku, Kyoto, 606-8501 Japan; 7 Center for the Development of Cognitive Behavior Therapy Training, Shirogane-cho 1-13, Shinjuku-ku, Tokyo, 162-0816 Japan

**Keywords:** Depressive symptom, Patient Health Questionnaire-9, Item response, Suicide ideation, National Health and Nutrition Examination Survey, Exponential distribution, Latent trait

## Abstract

**Background:**

Recently, item responses and total scores on depression screening scales have been reported to have characteristic distributions in the general population. The distributional pattern of responses to the Patient Health Questionnaire-9 (PHQ-9) in the general population has not been well studied. Thus, we carried out a pattern analysis of the PHQ-9 item responses and total scores in US adults.

**Methods:**

Data (5372 individuals) were drawn from the 2013–2014 National Health and Nutrition Examination Survey in the United States. The item responses and total score distributions of the PHQ-9 data were investigated with graphical analysis and exponential regression model.

**Results:**

Lines of item responses showed the same pattern among the nine items, characterized by crossing at a single point between “not at all” and “several days” and a parallel pattern from “several days” to “nearly every day” on a log-normal scale. The total score distribution of the PHQ-9 exhibited an exponential pattern, except for at the lower end of the distribution.

**Conclusions:**

The present results support that the item responses and total scores on the PHQ-9 in the general population show the same characteristic patterns, consistent with the previous studies using the Center for Epidemiologic Studies Depression Scale (CES-D) and Kessler Screening Scale for Psychological Distress (K6).

## Background

Depression is one of the leading causes of disability worldwide and contributes to a decreased functioning and diminished quality of life across a wide range of educational and socioeconomic levels [1]. Because the diagnosis of depression is based on the degree of depressive symptoms, there has been great interest in understanding the distribution of depressive symptoms in the general population [2]. To date, numerous population studies on depressive symptoms have been conducted using a variety of depression screening scales, such as the Center for Epidemiologic Studies Depression Scale (CES-D), the 6-item Kessler Screening Scale for Psychological Distress (K6), and Patient Health Questionnaire-9 (PHQ-9).

The CES-D is a forerunner of self-reported questionnaires for depressive symptoms in the general population and now serves as a screening tool in primary care and research settings [3]. The 20 items of the CES-D are grouped into the following two groups: 16 depressive symptoms and four positive affects (good, hopeful, happy, and enjoyed). The K6 was developed using item response theory and is used to measure the severity of psychological distress [4]. Although the K6 is a broad measure of psychological distress (depression, nervousness, restlessness, fatigue, worthlessness, and hopelessness), and it is widely used as a screening tool for major depression and anxiety disorders [5]. Finally, the PHQ-9 is one of the most widely used instruments for screening of clinical depression [6, 7]. The PHQ-9 reflects the nine criteria for major depression in the Diagnostic and Statistical Manual of Mental Disorders, Fifth Edition (DSM-5) [8]. There are overlapping items among the CES-D, K6, and PHQ-9; they include depressive symptom items such as “depressed,” “worthlessness” and “fatigue (tired)” in common.

Because the interpretations of a depression screening scale are clinically important, the optimal cut-off point for detecting major depressive disorder and the average scores in various populations have been investigated by many researchers [9, 10]. However, little attention has been paid to the distributional patterns of the item responses and total scores on the depression screening scales. The mathematical patterns of item responses and total scores are important for some reasons. If the mathematical patterns of item responses and total scores are established, they will be useful for predicting the frequency of individuals with a certain score in a population. Moreover, the mathematical patterns determine which statistical procedures to apply. If the empirical distributions of item responses and total scores follow a non-normal distribution, the statistical model of normal variables (e.g., parametric statistics) will require reconsideration [11]. Finally, if the distributions of item responses and total scores follow a specific mathematical pattern, this may hint the mechanism of depressive symptoms.

**Fig. 1:**
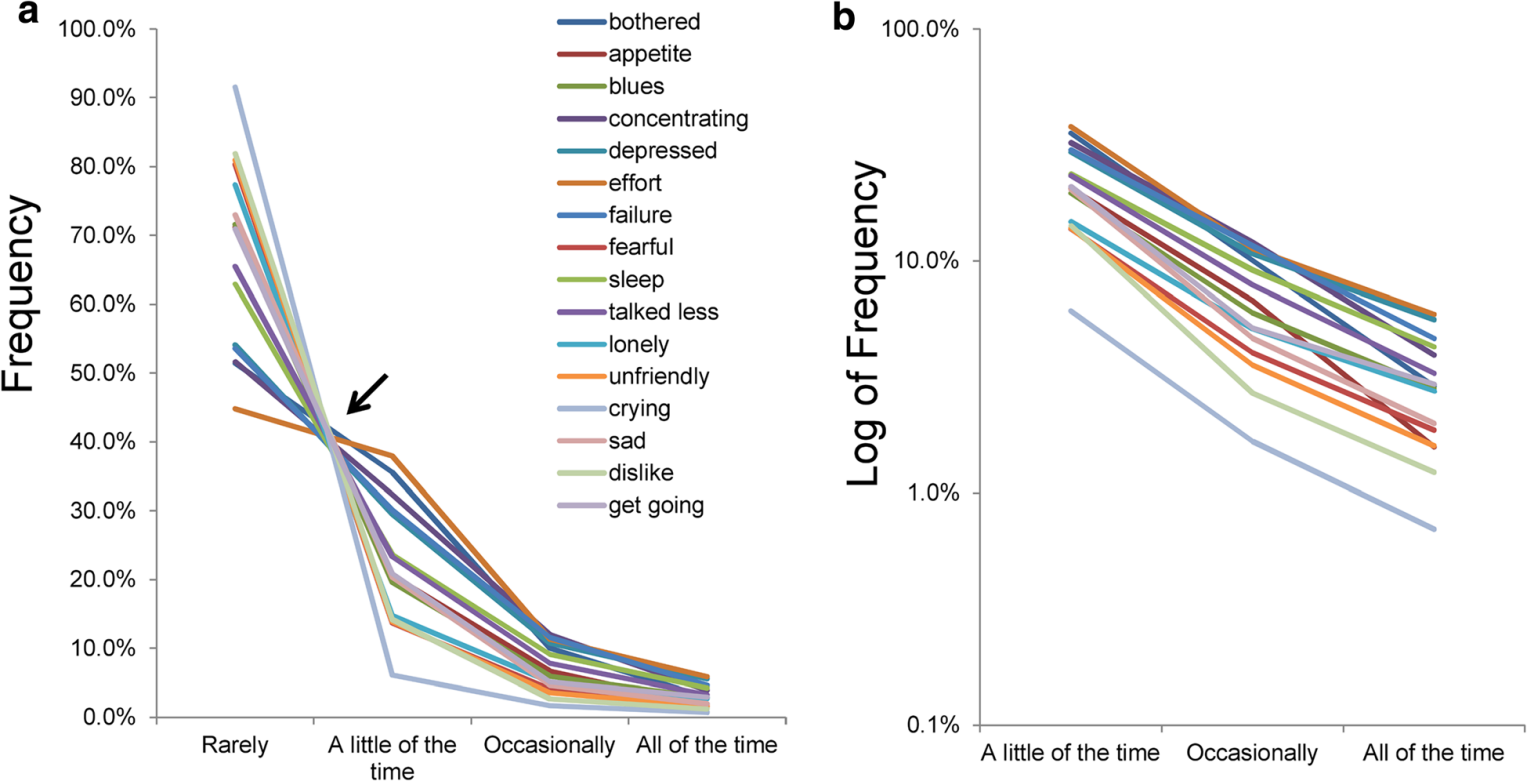
Item responses on the CES-D. Item responses to the 16 depressive symptom items are presented using a normal scale (**a**) and a log-normal scale (**b**). **a** The lines for the 16 items crossed between “rarely” and “a little of the time.” Between “a little of the time” and “all of the time,” the same lines decreased regularly. **b** The lines for the 16 items showed a parallel pattern between “a little of the time” and “all of the time” on a log-normal scale. Image credit: PLoS ONE at 10.1371/journal.pone.0165928.g001

Recently, analyzing the CES-D data from nearly 32,000 respondents of a Japanese national representative survey, we first reported that all item responses followed a similar pattern among the 16 depressive symptom items (Fig. 1) [12, 13]. The CES-D allows individuals to self-rate the amount of time of each depressive symptom during the past week, from “rarely,” “a little of the time,” “occasionally,” and “all of the time” [3]. As shown in Fig. 1, the lines for the 16 items cross at a single point between “rarely” and “a little of the time,” after which they decrease regularly (Fig. 1a). Using a log-normal scale, the lines of the item responses show a parallel decreasing pattern from “a little of the time” to “all of the time” (Fig. 1b) [12]. These item response patterns have been confirmed in an analysis of the CES-D data from 8000 Japanese employees [14] and the 6-item Kessler Screening Scale for Psychological Distress (K6) data from the National Survey of Midlife Development in the United States (MIDUS) [15].

In general, the response options of depression screening scales start with a negative adverb option at the lower end (e.g., never, rarely, and none), and continue with the degree adverb options for the remaining response options (e.g., a little, some, much, most, and all) [3, 16]. Mathematically, if the ratios of the adjacent degree adverb options are the same among all the items, the lines for item responses can cross at a single point between the negative adverb option and the adjacent degree adverb option, and they show a parallel pattern during the degree-adverb options on a log-normal scale. In fact, the ratios of the adjacent degree adverb options were similar among all items in the previous studies [12, 15].

Furthermore, the total scores on depression screening scales in the general population have been reported to approximate an exponential pattern except for the lower end of the distribution. These findings have been replicated in an analysis of Revised Clinical Interview Schedule (CIS-R) data from the British National Household Psychiatric Morbidity Survey [17], the CES-D data from the same nationally representative surveys [14, 18], and the K6 data from the MIDUS [15].

Taken together, these findings suggest that the item responses and total scores on depression screening scales follow the same characteristic pattern in the general population. The degree to which these findings can be generalized to other depression scales is unclear but warrants examination. To date, there are few studies that have investigated the distributional patterns of the item responses and total scores on the PHQ-9 in the general population. Thus, we investigated whether the item responses and total scores on the PHQ-9 follow characteristic patterns, consistent with other depression screening scales. If the empirical distributions of item responses and total scores on the PHQ-9 are shown to represent specific distributions, it will shed light on the mechanism of depressive symptoms. For example, the results of the previous factor analytic studies are incongruent about the number of latent traits of depressive symptoms [19]. If the distributions of depressive symptoms are proven to exhibit a common mathematical pattern, it may provide further evidence of the number of latent traits of depressive symptoms.

Moreover, the analysis of the PHQ-9 data potentially enables a further understanding of the prevalence of suicidal ideation in the general population. While the CES-D and K6 have no item about suicidal ideation, the PHQ-9 includes an item about suicidal ideation: “thoughts of being better off dead and active ideas of self-harm” [8]. While the prevalence of suicidal ideation is often expressed in percentage, the severity of suicidal ideation (intensity and duration of a patient’s thoughts about suicidal ideation) varies from individual to individual [20]. Furthermore, the risk of suicide death or suicide attempt increases with the severity of suicidal ideation [21–23]. Thus, to understand the prevalence of suicide ideation in the general population, it is necessary to elucidate the severity distribution of suicidal ideation. In this study, we sought to elucidate the pattern of response to the suicidal ideation item among the general population and determine whether the item response follows a characteristic pattern consistent with other items.

This study used the PHQ-9 data from the National Health and Nutrition Examination Survey (NHANES). The NHANES is a national survey conducted to understand the health and nutritional status of people in the United States [24], and the PHQ-9 has been included as part of the NHANES since 2006 [10]. The sample for the NHANES was designed to represent the US population and minimize selection bias. The PHQ-9 data from the NHANES are adequate to confirm the reproducibility of the findings due to the sufficiently large sample sizes. The NHANES data are accessible to researchers around the world and have been utilized in numerous studies on public health.

The aim of this study was to elucidate the patterns of item responses and total scores on the PHQ-9 in the general population and determine whether they follow the characteristic patterns consistent with the CES-D and K6. Furthermore, we investigated the pattern of item response of suicidal ideation and determined whether the item response of suicidal ideation item in the general population follow the characteristic pattern, consistent with depressive symptom items.

## Methods

### Dataset

Data were drawn from the 2013–2014 NHANES. De-identified data from the NHANES are openly available for researchers [24]. The 2013–2014 NHANES includes a nationally representative sample of non-institutionalized civilian US citizens selected with a four-stage sample design [24]. The first stage comprised primary sampling units from a frame of all US counties. NHANES primary sampling units were selected with probabilities proportional to a measure of size. The second stage consisted of a sample of area segments. The sample was arranged to represent approximately equal sample sizes per primary sampling unit. The third stage of sample selection comprised dwelling units. In a given primary sampling unit, a list of all dwelling units was arranged, and a subsample of these was arranged to screen potentially sampled individuals. The subsampling rates were allocated to constitute a national, equal probability sample of households. The fourth stage comprised individuals within occupied dwelling units or households. A list of all eligible persons within a household was prepared, and a subsample of participants was selected based on their sociodemographic indicators. To correct for sampling bias, NHANES oversamples people aged 60 years or over, African Americans, Asians, and Hispanics. The survey consisted of an individual interview and a health examination. Written consent was obtained from participants. In the 2013–2014 NHANES, 14,332 individuals were selected, 10,175 completed the interview, and 9813 were examined.

Data from participants aged 18 years and older were drawn from the 2013–2014 NHANES. The sample consisted of 5924 respondents (ages 18–19 years, *N* = 336 [male: *n* = 154]; ages 20–29 years, *N* = 919 [male: *n* = 453]; ages 30–39 years, *N* = 961 [male: *n* = 461]; ages 40–49 years, *N* = 1007 [male: *n* = 464]; ages 50–59 years, *N* = 916 [male: *n* = 443]; ages 60–69 years, *N* = 2253 [male: *n* = 1155]; ages 70–79 years, *N* = 535 [male: *n* = 253]; ages 80 years and older, *N* = 332 [male: *n* = 153]). Among the 5924 respondents, 42% (*N* = 2491) were non-Hispanic white, 20.6% (*N* = 1223) were non-Hispanic black, 14.0% (*N* = 830) were Mexican, 11.4% (*N* = 675) were Asian, 8.8% (*N* = 523) were other Hispanic races, and 3.1% (*N* = 182) were other races, including multi-racial. Among 5588 respondents aged 20 years and older, the responses to the highest grade or level of education were as follows: less than 9th grade (7.9%, *N* = 439), 9–11th grade (13.7%, *N* = 765), high school graduate (22.5%, *N* = 1257), some college degree (30.8%, *N* = 1722), and college graduate or above (25.1%, *N* = 1400). The sociodemographic characteristics of the 2013–2014 NHANES samples are reported in detail elsewhere [24].

### Ethics statement

The present study is a secondary analysis of freely available data. Since the ethics committee of the Panasonic Health Center does not regard de-identified secondary data analysis as research on human subjects, the ethics committee of the Panasonic Health Center waived the need for formal ethical approval and ruled that no formal approval was required for the present study.

### Measures

In the 2013–2014 NHANES, depressive symptoms were assessed using the PHQ-9. The nine items of the PHQ-9 reflect the nine criteria on which DSM-5 diagnoses of depressive disorders are based [25]. The PHQ-9 assesses the frequency of a variety of depressive symptoms within the past 2 weeks with 4-point response options: 0 = “not at all,” 1 = “several days,” 2 = “more than half the days,” and 3 = “nearly every day.” Total scores can range from 0 to 27.

### Analysis

First, we analyzed the distributions of the PHQ-9 item responses. Participants who did not respond to all PHQ-9 items (552 individuals) were excluded from the analysis. The non-response rate for each item (e.g., “refused,” “don’t know,” or “missing” to each item) was 8.98% (*N* = 532) for “anhedonia,” 9.00% (*N* = 533) for “depressed mood,” 8.93% (*N* = 529) for “sleep problems,” 8.93% (*N* = 529) for “low energy,” 8.95% (*N* = 530) for “appetite change,” 9.00% (*N* = 533) for “low self-esteem,” 8.98% (*N* = 532) for “concentration difficulties,” 8.98% (*N* = 532) for “psychomotor agitation or retardation,” and 9.00% (*N* = 533) for “suicidal ideation.” The final sample for the item response analysis comprised 5372 individuals.

Item response rates were calculated for all nine items. According to previous studies, all ratios of the degree adverb option to the adjacent degree adverb option are similar among depressive symptom items [12, 15]. Thus, the ratios of “more than half the days” to “several days” and “nearly every day” to “more than half the days” were calculated for all nine items. To evaluate the complex pattern of item responses, item responses were analyzed with histograms using normal and log-normal scales. According to the mathematical model of item responses in the previous study, if the ratios of the degree adverb option to the adjacent degree adverb option are similar among all items, all lines for the nine items will follow a parallel pattern on a log-normal scale [12]. As the pattern of item responses was different according to the response options, unitary regression analysis was not appropriate for the pattern analysis.

After confirming that the item of suicidal ideation followed the characteristic patterns observed in depressive symptom items, we analyzed the distribution of the PHQ-9 total scores. The pattern of total score distribution was analyzed by graphical analysis. Since an exponential pattern shows a linear pattern with a log-normal scale, a log-normal scale enables us to detect the extent of an exponential pattern. The least square method was used in regression analysis. Data were analyzed using JMP software, version 11 (SAS Institute Inc., Cary, NC, USA).

## Results

### PHQ-9 item responses

Table 1 displays the item responses of the PHQ-9. The item responses for all nine items showed a similar pattern, with the frequencies being the highest for “not at all,” decreasing from “not at all” to “more than half the days,” and being the lowest for “more than half the days” or “nearly every day.” Regarding item 9 (suicidal ideation), the percentages of item responses for “not at all,” “several days,” “more than half the days,” and “nearly every day” were 96.6, 2.3, 0.6, and 0.6%, respectively. There were no exceptions to this pattern. The ratios of “more than half the days” to “several days” (0.23 to 0.36) were smaller than those of “nearly every day” to “more than half the days” (0.77 to 1.42).

**Table 1:** PHQ-9 item responses

Item	Item response (%)				Rate of “2” to “1”	Rate of “3” to “2”
	0	1	2	3		
Anhedonia	73.8	16.3	5.4	4.4	0.33	0.81
Depressed mood	75.7	16.8	3.9	3.6	0.23	0.93
Sleep problems	63.3	20.9	6.5	9.2	0.31	1.42
Low energy	49.1	34.0	7.8	9.0	0.23	1.15
Appetite change	74.8	15.5	5.1	4.6	0.33	0.90
Low self-esteem	82.6	11.5	2.9	3.0	0.25	1.06
Concentration difficulties	82.1	10.9	3.6	3.5	0.33	0.98
Psychomotor agitation or retardation	88.8	6.8	2.5	1.9	0.36	0.77
Suicidal ideation	96.6	2.3	0.6	0.6	0.26	1.00
Average	76.3	15.0	4.3	4.4	0.29 ± 0.05	1.00 ± 0.20

Each of the nine items is scored with 4-point response options: 0 (*not at all*), 1 (*several days*), 2 (*more than half the days*), and 3 (*nearly every day*). Average rate data are presented as Mean ± 1SD

**Fig. 2:**
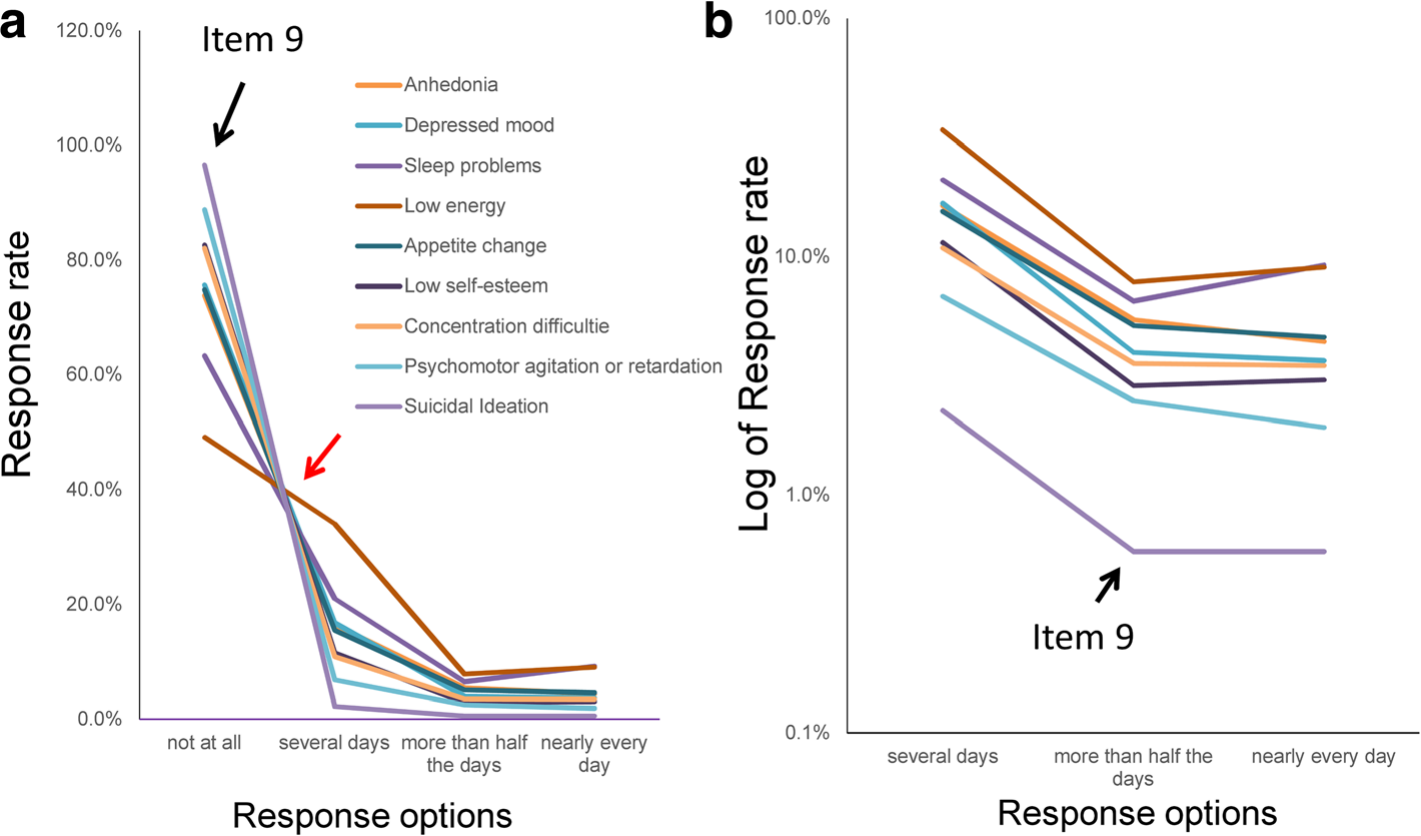
Item responses on the PHQ-9. **a** The item responses of the all items showed a common pattern of different types of distributions with a boundary at “several days.” Between “not at all” and “several days,” as indicated by the red arrow, the lines for the nine items crossed at a single point. Between “several days” and “nearly every day,” lines for the nine items seemed to barely cross each other. As indicated by the black arrow, the line for item 9 followed the same pattern as those of other depressive symptom items. **b** Using a log-normal scale, lines of the item responses showed a parallel pattern from “several days” to “nearly every day.” The gradient of the linear pattern of item responses moved downward between “several days” and “more than half the days” (blue arrow) and horizontally between “more than half the days” and “nearly every day” (red arrow). As indicated by the black arrow, although the line for item 9 was a little away from other lines, it was almost parallel to those of other depressive symptom items

To compare the complex pattern of the item responses, we plotted all item responses on the same chart (Fig. 2). The item responses of the all items exhibited a same mathematical distribution, which showed different types of patterns with “several days” forming a boundary (Fig. 2a). Between “not at all” and “several days,” as pointed by the red arrow in Fig. 2a, the lines for the nine items crossed at a single point. On the other hand, between “several days” and “nearly every day,” lines for the nine items appeared to barely cross each other.

Using a log-normal scale, lines of the item responses showed a parallel pattern between “several days” and “nearly every day” (Fig. 2b). The gradient of the linear pattern of item responses moved downward from “several days” to “more than half the days” (blue arrow) and horizontally from “more than half the days” to “nearly every day” (red arrow). These observations are consistent with the ratios of “more than half the days” to “several days” (0.23 to 0.36) being smaller compared to those of “nearly every day” to “more than half the days” (0.77 to 1.42). The degree of parallelism of the nine lines represents the similarity of the ratios of “more than half the days” to “several days,” and “nearly every day” to “more than half the days” among the nine items. Although the line for Item 9 was a little away from the other lines, it was almost parallel to those of other depressive symptom items.

### Total score distribution of the PHQ-9

**Fig. 3:**
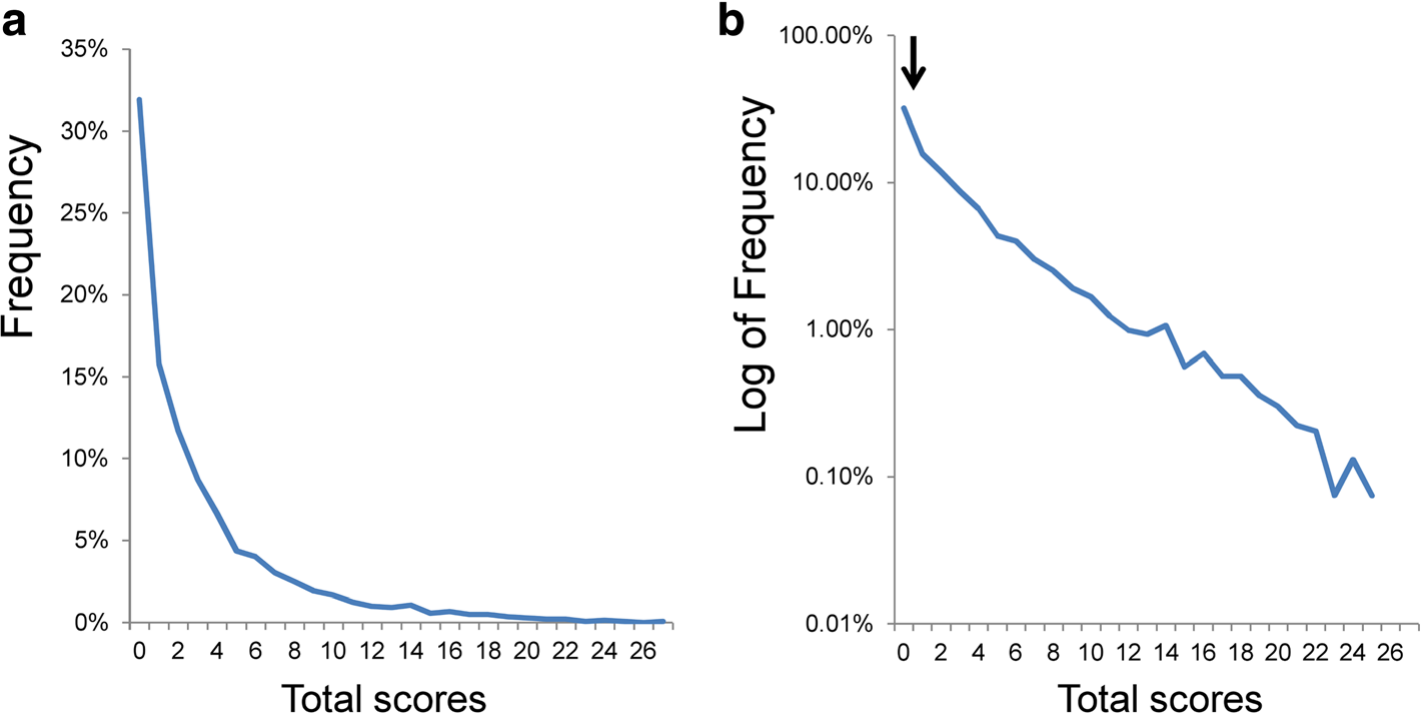
Total score distribution of the PHQ-9. **a** The total score distribution was right-skewed, and the frequency of the zero score was 31.9%. **b** Using the log-normal scale, the total score distribution showed a linear pattern. As indicated by the arrow, the total score distribution exhibited slightly higher frequencies compared to those predicted from the exponential pattern at the lower end of the distributions

Figure 3a depicts the total score distribution of the PHQ-9. The total score distribution was right-skewed, and the frequency of the zero score was 31.9%. Using the log-normal scale, the total score distribution showed a linear pattern, suggesting that the total PHQ-9 scores approximated an exponential pattern (Fig. 3b). As indicated by the arrow, the total score distribution exhibited slightly higher frequencies compared to those predicted from the exponential pattern at the lower end of the distributions.

A regression curve for the exponential model was calculated from 0 to 25 points (*y* = 0.20e^-0.211x^, *R*^2^ = 0.98). The independent variable (x) was the total score, and the dependent variable (y) was the percentage of participants. *R*^2^ was the coefficient of determination. The analysis indicated a high coefficient of determination, confirming that an exponential distribution was a good fit to the total PHQ-9 scores.

## Discussion

The main findings were as follows: (1) the item responses and total scores on the PHQ-9 showed characteristic patterns, consistent with other depression screening scales; and (2) the response to item 9 of the PHQ-9 showed the same pattern as other depressive symptom items.

### Item response patterns on the PHQ-9

The item responses on the PHQ-9 exhibited the same mathematical pattern as the other depression screening scales, such as the CES-D and K6 [12, 15]. The pattern of the item responses was characterized by crossing at a single point between “not at all” and “several days” and a parallel pattern from “several days” to “nearly every day” on a log-normal scale. As noted in the Introduction, the characteristics of such an item response pattern could be explained mathematically [12]. If the ratios of “more than half the days” to “several days” and “nearly every day” to “more than half the days” are similar among all items, the lines for item responses exhibit the characteristic pattern [12]. In fact, the ratio of “more than half the days” to “several days” and “nearly every day” to “more than half the days” were similar among all the items. Further research is needed to clarify how the ratios of the adjacent degree adverb options on depression screening scales are similar among all items.

In the present study, the average ratios of “several days” to “more than half the days” (0.29) and “more than half the days” to “nearly every day” (1.00) differed according to the level of item responses (Table 1). According to the finding that boundary curves of each depressive symptom score in the distribution of total depressive symptom scores approximate an exponential pattern [26], the probability of each degree adverb option reflects the “distance” for each degree adverb option. Thus, it is suggested that the distance of “more than half the days” is approximately equal to that of “nearly every day” and one-third of that of “several days.”

The results of this study show that the response to item 9 exhibited the same pattern as the responses to the other eight depressive symptom items, suggesting that suicidal ideation shares a common mechanism with the other eight depressive symptoms. From a neuroscientific standpoint, the common mechanism of depressive symptoms is comprehensible. Many researchers have suggested that specific neural regions constitute a final common pathway for depressive symptoms [27, 28], suggesting that all depressive symptoms are influenced by the activity of this final common pathway.

Although the response to item 9 can identify individuals at an increased risk of suicide ideation, screening a given individual’s suicide attempt possibility is another question. In general, it is not easy to predict individual patient-level suicide [23]. Simon et al. reported that the risk of a non-fatal or fatal suicide attempt over 1 year increases from about 0.4% among outpatients reporting suicidal ideation of “not at all” to 4% among those reporting suicidal ideation “nearly every day” [29]. Of note, although the outpatients reporting suicidal ideation “nearly every day” have a 10 times higher risk of a non-fatal or fatal suicide attempt than those reporting suicidal ideation “not at all,” 96% of outpatients reporting suicidal ideation “nearly every day” do not commit suicide over 1 year, indicating the difficulty in screening for potential suicidality [22, 23, 29]. We can use responses to item 9 as a predictor of individual patient-level suicide but making an accurate prediction/representation of suicidality is not possible. The low specificity of item 9 to screen for acute suicide could be partly explained how the item is worded [30]. Item 9 consists of two parts: thoughts of being better off dead and thoughts of hurting oneself in some way. However, most patients who endorse item 9 appear to be agreeing with the first part only [31, 32]. Consequently, although the response to item 9 has the potential to identify individuals at increased risk of suicide, further procedures would be necessary to assess the actual risk of suicide in individuals endorsing Item 9.

### Distribution of the total score on the PHQ-9

Our findings indicate that the total PHQ-9 score from the NHANES data exhibits an exponential pattern except at the lower end of the distribution. These findings are consistent with results of other nationally representative surveys that the K6, CIS-R, and CES-D in the USA, England, and Japan, respectively [15, 17, 18]. Previous research demonstrated that the response rate of the negative adverb option (“not at all”) plays an important role in predicting the non-exponential pattern at the lower end of the distribution [13, 33]. Specifically, at the lower end of the distribution, the sum of the item scores with a high rate for the negative adverb option showed higher scores in comparison with those expected from the exponential pattern. Conversely, the sum of item scores with a low rate for the negative adverb option showed lower scores. Actually, according to our calculations, the average probability of “not at all” in the present study (76.3%) was higher than that of the MIDUS data (61.8%), which represented an exponential distribution for the whole extent of total scores [15].

There are limitations to this study. Though we investigated whether item responses and total item scores on the PHQ-9 followed characteristic patterns observed in other studies, we did not evaluate the fitness of other mathematical models. Given that item responses and total item scores showed exponential-related patterns, we could not propose other mathematical models. Future research might seek to investigate the comparative fit of other models to the NHANES data. Additionally, although we assessed the similarity of the pattern of item responses through graphical analysis, the similarity of the pattern was not quantified due to the complexity of the pattern. A simple and unitary pattern allows for applying existing distribution models (Gaussian models, exponential models, etc.) and calculating the goodness of fit through unitary regression models. Conversely, in the case of such a complex pattern, these procedures are difficult. Further research is needed to develop a quantification method of the similarities between the complex patterns.

However, our study has methodological advantages. Although the method of the present study was simple (visualization with histograms), it enabled the identification of a complex pattern of item responses. Graphical analysis is essential for exploratory data analysis of complex models [34, 35]. In addition, as noted in the Introduction, using data from the NHANES ensured a large sample size with limited selection bias. Moreover, since all data were publicly available, researchers can easily review the present findings using the raw data.

In the end, evidence that item responses and total scores on the PHQ-9, CES-D, and K6 follow the same mathematical patterns will shed light on the process of depressive symptomatology. In general, depressive symptoms are measured with depression screening scales and these total scores are clinically used as an index of depression severity. To support such procedures, all depression symptoms must constitute a single latent trait [36]. However, previous studies using factor analysis contradict regarding the number of latent traits in depression screening scales [19]. The findings of this research could complement those of previous studies. Based on the same mathematical pattern of item responses among all nine items, all depressive symptoms may share a common pathway. Further research should be carried out to clarify the mechanism of the mathematical patterns in depression rating scales in the general population. Moreover, much of the research up to now has been limited to the data from Japan, the US, and the UK. The degree to which these results generalize to other countries is unclear and, therefore, warrants examination.

## Conclusions

The results of this study support that the item responses and total scores on the PHQ-9 follow the characteristic distributions, consistent with other depression screening scales. Given that item responses show the same pattern among all nine items, we conjecture that all nine items share a common pathway. The findings of this research extend our knowledge of depressive symptomatology and will serve as a basis for the estimation of how depressive symptoms are distributed in a general population.

## Abbreviations

CES-D: Center for Epidemiologic Studies Depression Scale
CIS-R: Revised Clinical Interview Schedule
K6: Kessler Screening Scale for Psychological Distress
MIDUS: National Survey of Midlife Development in the United States
NHANES: National Health and Nutrition Examination Survey
NHIS: National Health Interview Survey
PHQ: Patient Health Questionnaire
